# Does concurrent adenoidectomy or tonsillectomy affect the graft success rate of cartilage myringoplasty in adults?

**DOI:** 10.1186/s12893-021-01283-3

**Published:** 2021-06-08

**Authors:** Zhengcai Lou

**Affiliations:** Department of Otorhinolaryngology, Yiwu Central Hospital, 699 Jiangdong Road, Yiwu, 322000 Zhejiang China

**Keywords:** Endoscope, Myringoplasty, Adenoidectomy, Tonsillectomy, Graft success

## Abstract

**Background:**

The objective of this study was to evaluate the graft success and hearing outcomes of concurrent adenoidectomy or tonsillectomy and myringoplasty.

**Methods:**

Medical case notes were reviewed for all adult patients with dry perforations who had undergone myringoplasty, with or without concurrent throat surgery, from December 2015 to February 2018. The study population was divided into concurrent myringoplasty and throat surgery (Group A) and single myringoplasty (Group B) groups. The air–bone gap (ABG) and graft success rate were evaluated in both groups.

**Results:**

A total of 131 ears of 131 patients were included in this study. In total, 33 ears of 33 patients were assigned to Group A and 98 to Group B. Of the 33 patients in Group A, adenoid residue was detected in 3, chronic tonsillitis in 21, and tonsil hypertrophy in 9. The graft success rate was 96.9 % in Group A and 96.9 % in Group B at 6 months postoperatively (p = 0.993). In addition, the graft success rate was 87.9 % in Group A and 92.8 % in Group B at 24 months postoperatively (p = 0.372). Reperforation occurred in three patients in Group A and four in Group B; the difference was not significant. No significant group differences were observed in preoperative (p = 0.654) or postoperative (p = 0.791) ABG values or mean ABG gain (p = 0.439). No patient in either group developed cholesteatoma of the middle ear.

**Conclusions:**

Simultaneous adenoidectomy or tonsillectomy and myringoplasty is feasible but does not improve the graft success rate or hearing outcome.

## Background

Chronic tympanic membrane (TM) perforation with chronic otitis media (COM) are related to inadequate ventilation through the eustachian tube (ET) [1, 2]. Adult patients with chronic otologic disease frequently have coexisting nasal and throat pathology, which can cause or worsen middle ear problems secondary to eustachian tube dysfunction (ETD) [1]. The causes of ETD include upper respiratory tract infection, sinusitis, allergic rhinitis, adenoid, tonsil hypertrophy, nasopharyngeal mass, cleft palate, and nasal septal deviation [1, 3−6]. ETD can result in chronic negative middle ear pressure, which can cause TM retraction, COM with effusion, and middle ear infection [2]. Thus, some studies suggested that nasal abnormalities and pathology should be identified and corrected before myringoplasty is performed [7, 8]. Sinonasal and nasopharyngeal procedures may improve ET function and, by extension, otologic outcomes [1].

Whether simultaneous ear and nasal/sinus procedures should be performed is debated. Two studies suggested that simultaneous myringoplasty and septoplasty is feasible in adults with both middle ear and sinonasal pathology, and is attractive in terms of operative and anesthetic morbidity, time, and the lower cost of surgically repairing nasal and ear problems simultaneously [1, 9]. However, others disapproved of simultaneous nasal surgery and myringoplasty because it increases the risk of graft reperforation [7, 10, 11]. Salvinelli et al. [12] recommended that tympanoplasty and nasal surgery not be performed at the same time, and that middle ear surgery should be carried out when the anatomy and physiology of the nasal, pharyngeal, and tubal mucosae have returned to normal. In children, Becker and Opitz [13] also concluded that adenoidectomy should not be performed concurrently with tympanoplasty because of frequent postoperative negative middle ear pressure. Charlett et al. [14] suggested that adenoidectomy before pediatric myringoplasty does not increase the likelihood of a successful outcome. However, few studies have evaluated the effect of throat disorders on the success of myringoplasty in adults. We evaluated the graft success rate and hearing outcome of concurrent adenoidectomy or tonsillectomy and myringoplasty.

## Methods

### Patients and methods

Medical case notes were reviewed for all adult patients with dry perforations who had undergone myringoplasty, with or without concurrent throat surgery, from December 2015 to February 2018 in a single teaching hospital. Exclusion criteria were the presence of cholesteatoma, malignant laryngeal tumors, revision cases, procedures involving ossicular reconstruction or mastoid surgery, history of previous adenoidectomy or tonsillectomy, and failure to attend postoperative follow-up. All operations were performed by the same surgeon.The perforation was classified according to size, as large (> 50 % of the eardrum), medium (25–50 %), or small (< 25 %).

The study population was divided into concurrent myringoplasty and throat surgery (Group A) and single myringoplasty (Group B) groups. The throat surgery group comprised patients who had a concurrent adenoidectomy or tonsillectomy. Data on age, sex, side, size of perforation, myringosclerosis, smoking status, follow-up duration, audiologic test results, TM graft status at the most recent follow-up, and surgical outcomes were obtained from the patient’s medical charts. Pure-tone audiometry (PTA) was performed preoperatively and at 12 months after surgery. Standard PTA was performed at the frequencies of 0.5, 1, 2, and 3 kHz. The air–bone gap (ABG) was calculated as the mean difference between air conduction and bone conduction at each frequency.

## Surgical techniques

### Concurrent myringoplasty and throat surgery (Group A)

**Fig. 1:**
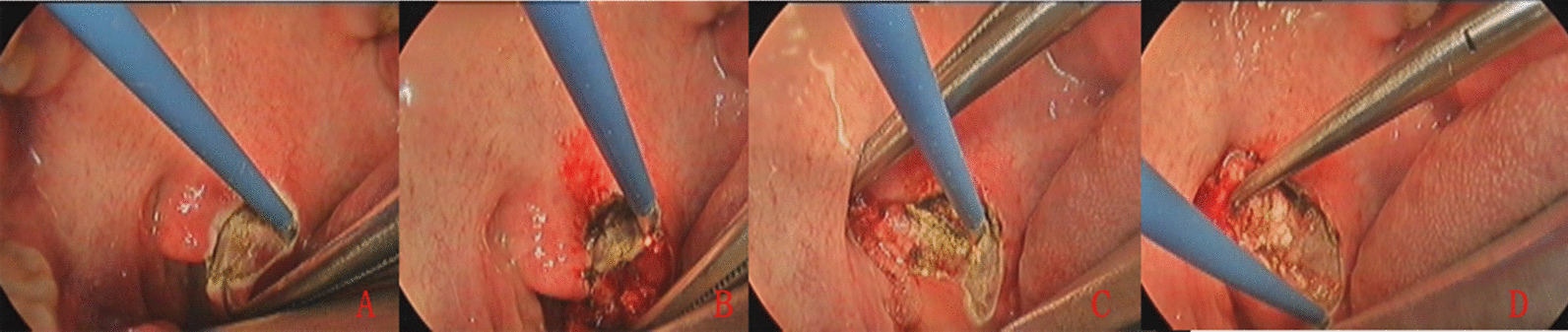
Photographs showing the upper pole of the tonsil (**A** and **B**), tonsils separated in peritonsillar space (**C**), and dissection of the inferior pole of the tonsil (**D**)

All patients were scheduled for simultaneous myringoplasty and tonsillectomy or adenoidectomy under general anesthesia. Same-day myringoplasty was performed in patients with bilateral TM perforations. The pharyngeal surgery was performed before myringoplasty. Tonsillectomies were performed using monopolar electrosurgery in patients with tonsil hypertrophy (Fig. 1), and plasma radiofrequency ablation was used for adenoidectomy. All tissue samples were sent for histological examination.

Following the tonsillectomy or laryngeal surgery, myringoplasty using a full-thickness cartilage graft was performed under a 0°, 4 mm ⋅ 18 cm rigid endoscope using the “push-through” technique. Cartilage from a single-layer perichondrium graft was harvested through a 1-cm incision medial to the ipsilateral tragus. The graft was 1–2 mm wider than the diameter of the perforation and was not thinned. If the malleus was exposed, a notch was created in the cartilage (but not the perichondrium) to accommodate the handle. The perichondrium lateral to the notch was peeled and elevated, thus becoming a patch of free perichondrium 2 mm wider than the notch. The composite graft was pushed through the perforation and placed medial to the remnant TM and the annulus in an underlay manner. Then, the cartilage notch was clipped to the malleus, and the patch of free perichondrium above the notch was placed lateral to the handle of the malleus. The tympanomeatal flap was not elevated in any patient. Biodegradable Nasopore soaked in antibiotic ointment was used to support the graft medially and laterally. The external auditory canal was packed with gauze soaked in antibiotic ointment up to the tragus incision, which was not sutured.

### Single myringoplasty group (Group B)

Single myringoplasty without tonsillectomy or adenoidectomy was performed. The surgical procedure for myringoplasty was similar to that for Group A.

### Postoperative follow-up

The patients were discharged after 2 days. The packing gauze soaked in antibiotic ointment was removed from the EAC at 2 weeks postoperatively, and the biodegradable Nasopore fragments were aspirated from the EAC at 3 weeks after surgery to allow visualization of the graft. All patients were scheduled for regular follow-up visits at 2 and 3 weeks and 1, 3, 6, 12, and 24 months after surgery in the otolaryngology outpatient clinic. Endoscopy was performed at all postoperative visits, and PTA was performed at the 12-month follow-up visit. Graft success was defined as the presence of an intact graft, as evaluated using a 0° endoscope. Graft failure was defined as residual perforation, recurrent perforation, graft lateralization, significant blunting, and medialization at 6 and 24 months postoperatively.

### Statistical analysis

Statistical analyses were performed using statistic package for social science (SPSS) software ver. 21.0; (SPSS Inc.,, Chicago, IL, USA). Data are expressed as means with standard deviations, or as percentages (%). The chi-squared test was used to compare categorical data. The Wilcoxon and Mann–Whitney U tests were employed to compare non-parametric variables, and the independent and paired samples *t*-tests were used to compare parametric variables. A p-value < 0.05 was considered to indicate statistical significance.

## Results

### Demographic data

**Fig. 2:**
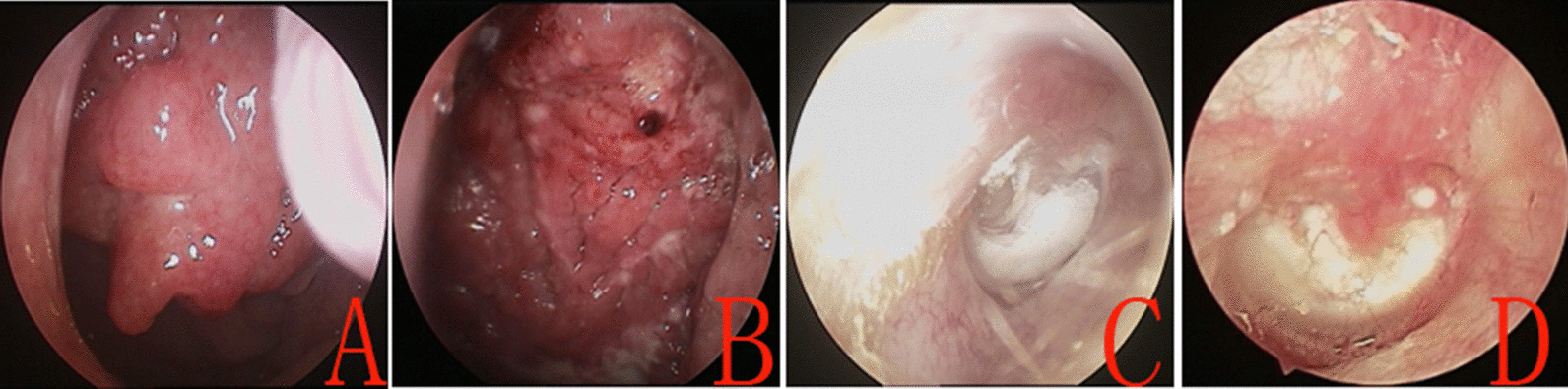
A-29-year-old male with right TM perforation and coexistent adenoid residue. The adenoid residue preoperatively (**A**), the nasopharynx at 2 weeks postoperatively (**B**), te preoperative perforation (**C**), and perforation closure at 3 months postoperatively (**D**)

In total, 131 ears of 131 patients met the inclusion criteria. Of them, 33 ears of 33 patients were assigned to the concurrent myringoplasty and throat surgery group (Group A), and 98 ears to the single myringoplasty group (Group B). Of the 33 patients in Group A, adenoid residue was detected in 3 patients (Fig. 2), chronic tonsillitis in 21, and tonsil hypertrophy in 9 (Fig. 1). The age, sex, side, type, size of perforation, myringosclerosis, and smoking status were matched between the two groups (Table 1). Postoperative pathology tests confirmed tonsil hypertrophy or adenoid in all cases in Group A. No evidence of postoperative bleeding was found.

**Table.1:** Demographic characteristic of patients between the groups

	A group	B group	P value
No	33	98	
Sex (F:M)	11:22	41:57	0.388^a^
Age (years)	47.6 ± 3.76	49.3 ± 4.17	0.573^b^
Side of ear (L:R)	20:13	69:29	0.297^a^
Size of perforation (Large: Medium:small)	9: 22: 2	28:59:11	0.391^a^
Type of perforation (marginal:central)	14:19	41:57	0.953^a^
Myringosclerosis (Y:N)	10:23	27:71	0.761^a^
Smoking status (Y:N)	7:26	16:82	0.523^a^
Graft success rate			
At postoperative 6^th^ months (N,%)	32 (96.9%)	95 (96.9%)	0.993^a^
At postoperative 24^th^ months (N,%)	29 (87.9%)	91 (92.8%)	0.372^a^
Re-perforation (N, %)	3(9.4%)	4 (4.21%)	0.268^a^

^a^Chi-square test

^b^Independent Samples Test

### Graft uptake rate and complications

**Fig. 3:**
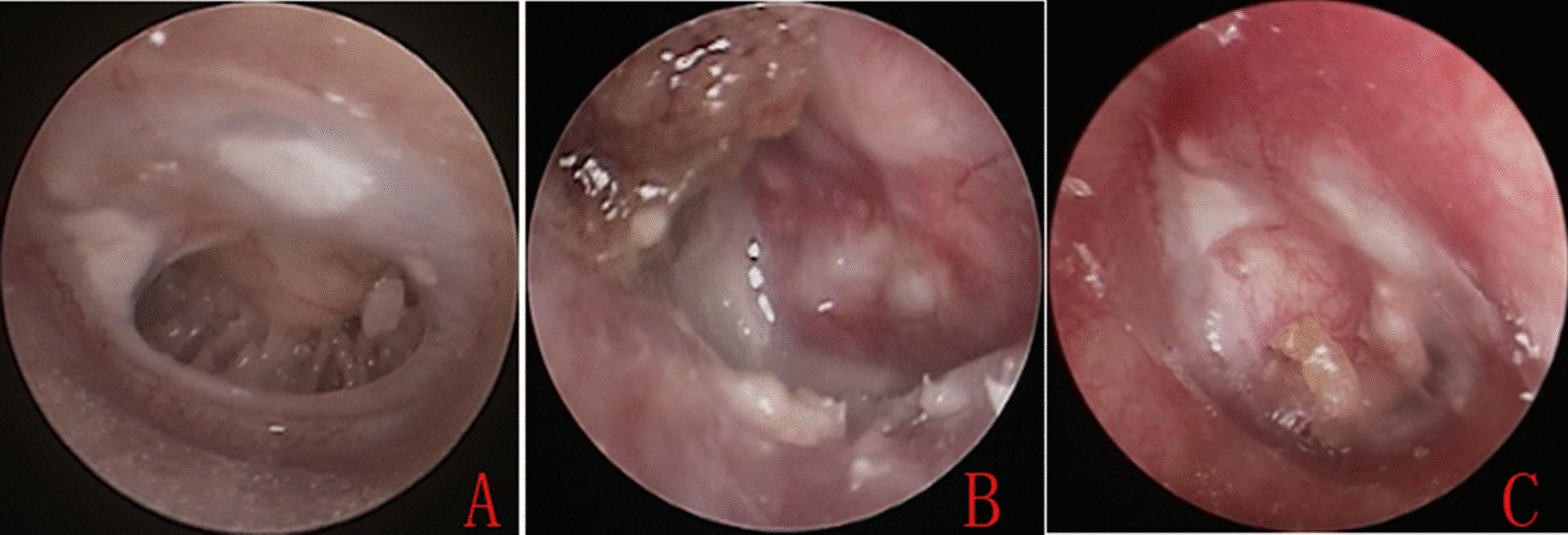
Photographs showing the perforation before surgery (**A**), and at 2 weeks (**B**) and 5 weeks (**C**) post-surgery (same patient as in Fig. 1)

All patients were followed up for 24 months. The graft success rate was 96.9 % (32/33) in Group A and 96.9 % (95/98) in Group B at 6 months postoperatively (p = 0.993) (Fig. 3). In Group A, one ear had postoperative purulent otorrhea and secondary middle ear infection, resulting in residual perforation. In Group B, residual perforation was seen in three patients. The graft success rate was 87.9 % (29/33) in Group A and 92.8 % (91/98) in Group B at 24 months postoperatively (p = 0.372). Reperforation occurred in three patients in Group A and four patients in Group B (p = 0.268) (Table 1). During follow-up, no adenoidectomy- or tonsillectomy-related complications were observed. No complications (iatrogenic sensorineural hearing loss, facial nerve palsy, vertigo, or tinnitus) were observed, and no graft lateralization or medialization, or significant blunting was noted. No patients in either group developed cholesteatoma of the middle ear.

### Hearing gain

In Group A, the mean pre- and postoperative ABGs were 26.83 ± 4.15 and 9.61 ± 2.54 dB at 12 months postoperatively (p = 0.001). In Group B, the values were 27.31 ± 6.91 dB and 8.74 ± 3.61 dB (p = 0.001). No significant group differences were observed in the preoperative (p = 0.654) or postoperative (p = 0.791) ABG values or mean ABG gain (p = 0.439) (Table 2). The functional success rate (postoperative ABG ≤ 20 dB) was 84.8 % (n = 28) in Group A and 87.8 % (n = 86) in Group B (p = 0.769).

**Table 2:** Comparison of hearing gains and the air-bone gap ((dB) mean ± SD)

	Pre-ABG	Post- ABG	P^1^	Gain (mean)	Hearing success (ABG ≤ 20 dB)
Group A (n = 33)	26.83 ± 4.15	9.17 ± 2.26	0.001*	17.41 ± 5.64	28 (84.8 %)
Group B (n = 98)	27.31 ± 6.91	8.74 ± 3.61	0.001*	19.31 ± 2.31	86 (87.8 %)
P^2^	0.654	0.791		0.439	0.769^a^

^1^Paired Samples test, ^2^Mann Whitney U test, ^a^Chi-square test

*p < 0.01

^1^Comparison ABG between the same groups pre- and postoperatively

^2^Comparison between two groups in terms of gain, pre- or postoperatively

## Discussion

ETD and the consequent hypoventilation of the middle ear are among the most frequent causes of failure of middle ear surgery. However, nasal or pharyngeal pathology is often thought to be responsible for inadequate tubal function. Therefore, potential interactions among the middle ear mucosa, ET function, and nasal or pharyngeal pathology are considered when planning myringoplasty [7, 8]. Several sinonasal and nasopharyngeal procedures can enhance tubal function and thereby improve otologic outcomes [8, 15, 16]. In addition, performing an adenoidectomy before TM reconstruction would improve graft survival rates for patients with adenoidal or tonsil hypertrophy [14]. However, the timing of sinonasal or nasopharyngeal procedures and myringoplasty are controversial. In most studies, myringoplasty was performed prior to a sinonasal or nasopharyngeal procedure, because transient tubal dysfunction and negative middle ear pressure can result in graft failure [7, 10]. However, in two studies simultaneous nasal surgery and myringoplasty did not affect the graft success rate [1, 9. Similarly, some studies reported frequent negative middle ear pressure in children following adenoidectomy, and this procedure should not be performed concurrently with tympanoplasty [13, 14].

in this study, the graft success rate was 96.9 % (32/33) in Group A and 96.9 % (95/98) in Group B at 6 months postoperatively (p = 0.993) (Fig. 3). In Group A, one ear had postoperative purulent otorrhea and secondary middle ear infection, resulting in residual perforation. In Group B, residual perforation was seen in three patients. The graft success rate was 87.9 % (29/33) in Group A and 92.8 % (91/98) in Group B at 24 months postoperatively (p = 0.372). No significant group differences were observed in the preoperative (p = 0.654) or postoperative (p = 0.791) ABG values or mean ABG gain (p = 0.439).

Our data indicate that simultaneous myringoplasty and throat surgery is efficacious in terms of TM graft survival and overall surgical success. These results are in agreement with Schuman, who concluded that simultaneous tympanoplasty and nasal surgery is feasible in adults [1]. Also, simultaneous surgery is attractive in terms of operative and anesthetic morbidity, time, and cost.

Adenoidectomy and tonsillectomy did not improve the graft success rate in patients with adenoid residue and tonsil hypertrophy, respectively, as reported previously in children [13, 14]. Charlett et al. [14] found that adenoidectomy before pediatric myringoplasty may not improve the likelihood of a successful outcome. Interestingly, Vartiainen et al. [17] performed a retrospective study of 60 pediatric patients with dry TM perforation undergoing type I tympanoplasty and found that all failures occurred in patients who had previously undergone adenoidectomy or adenotonsillectomy [17]. One possible explanation for this is that long-term adenoid or tonsillar hypertrophy resulted in morphological changes and irreversible stenosis of the cartilaginous part of the ET, but not of the edema of the mucous membrane at the tubal orifice. This precludes normalization of ET morphology even if the adenoid or tonsil is removed. Becker et al. [13] reported that most ET functions had not returned to normal following adento-tonsillectomy in children. In addition, rhinoplasty did not improve the function of the ET or the outcome of myringoplasty [7, 18, 19].

Although passive tubal parameters showed considerable improvement in many patients, there was no clear improvement of active tubal parameters following nasal surgery in most patients [7]. In contrast, coexisting chronic sinusitis is the factor most significantly associated with graft failure and reperforation [20]. Therefore, it has been suggested that sinonasal and nasopharyngeal procedures are useful for improving ET function in cases with chronic nasal or nasopharyngeal infection, if accompanied by poor tubal function [7]. A possible mechanism is direct inflammation arising from chronic infection at the tubal orifice on the mucous membrane. Sinusitis, and its irritant effect on the tubal mucous membrane, may resolve following surgery [8]. Other studies reported that negative middle ear pressure is related to graft retraction rather than failure [8]. In addition, cartilage grafts are stiff and can easily withstand negative middle ear pressure, which may have contribute to the development of otitis media and significantly affect postoperative healing outcomes [21, 22]. Therefore, cartilage grafts may prevent changes in ET function after myringoplasty.

This study was limited by the lack of assessment of preoperative and postoperative ET function. Also, this was not a randomized controlled trial, and multivariate analysis was not performed to identify risk factors for graft failure.

## Conclusions

Concurrent adenoidectomy or tonsillectomy and myringoplasty is feasible but does not improve the graft success rate or hearing outcome.

## Data Availability

All data generated or analyzed during this study are included in the published article.

## Abbreviations

TM: Tympanic membrane
COM: Chronic otitis media
ET: Eustachian tube
ETD: Eustachian tube dysfunction
ABG: Air-bone gap
SPSS: Statistic package for social science
